# Multidisciplinary Management of Massive Pulmonary Embolism in Twin Pregnancy Using Percutaneous Thrombectomy: Maternal-Fetal Survival

**DOI:** 10.7759/cureus.105400

**Published:** 2026-03-17

**Authors:** Alma Celeste Avilés Castillo, Rolando Jiron, María Esther Suárez García, Milton J Valdez, Rodrigo Gonzalez, Mauricio A Manzanarez Balladares, Maria L Lopez, Christopher Kaleb Romero Ríos

**Affiliations:** 1 Obstetrics and Gynaecology, Hospital Militar Escuela "Dr. Alejandro Dávila Bolaños", Managua, NIC; 2 Interventional Cardiology, Hospital Militar Escuela "Dr. Alejandro Dávila Bolaños", Managua, NIC; 3 Internal Medicine and Critical Care, Hospital Militar Escuela "Dr. Alejandro Dávila Bolaños", Managua, NIC; 4 Medicine, Hospital Militar Escuela "Dr. Alejandro Dávila Bolaños", Managua, NIC

**Keywords:** anticoagulation, case report, massive pulmonary embolism, obstructive shock, percutaneous mechanical thrombectomy, preterm labor, twin pregnancy

## Abstract

Massive pulmonary embolism (PE) during pregnancy is a major cause of maternal mortality, with increased risk in twin gestations. Hemodynamic instability in the setting of contraindication to systemic thrombolysis poses a critical therapeutic challenge, requiring a multidisciplinary approach to ensure maternal stabilization and fetal viability. A 32-year-old woman at 29 weeks of twin pregnancy was admitted with bilateral massive PE and obstructive shock. Due to the high obstetric hemorrhagic risk associated with systemic thrombolysis, percutaneous mechanical thrombectomy was performed. The procedure achieved pulmonary reperfusion and reversal of right ventricular dysfunction, allowing maternal stabilization and short-term prolongation of the pregnancy. Subsequently, cesarean delivery was performed due to preterm labor, resulting in two live newborns (Apgar scores of 8/9). The mother had an uneventful recovery without hemorrhagic complications. Massive PE in twin pregnancy can be successfully managed through a multidisciplinary strategy focused on maternal-fetal safety. In this context, percutaneous mechanical thrombectomy represents a safe and effective therapeutic alternative, associated with favorable maternal and perinatal outcomes.

## Introduction

Massive pulmonary embolism (PE) during pregnancy constitutes a life-threatening obstetric emergency and remains one of the leading direct causes of maternal mortality in developed countries, with an estimated incidence of one in 1,500 deliveries [1]. Pregnancy itself induces a physiological hypercoagulable state; however, this condition is further amplified in twin gestations. Recent studies indicate that multiple pregnancy carries up to a fourfold increased risk of severe maternal morbidity and thromboembolic events compared to singleton pregnancies, due to increased venous stasis from uterine compression and elevated production of procoagulant factors [2]. The management of PE during pregnancy represents a critical obstetric dilemma. Although systemic anticoagulation is the initial standard of care, hemodynamic instability requires immediate reperfusion therapy. Systemic thrombolysis, the treatment of choice in the general population, is associated in pregnancy with concerning rates of major bleeding (up to 6-18%) and risk of placental abruption, posing significant fetal risk [3].

In this scenario, the most recent clinical guidelines (2024) and Pulmonary Embolism Response Teams (PERT) have begun positioning catheter-directed therapies, specifically percutaneous mechanical thrombectomy, as a safe and effective rescue alternative [4]. This technique allows rapid relief of right ventricular outflow obstruction with a superior safety profile by minimizing systemic bleeding risk [4]. We present the case of a 29-week twin pregnancy complicated by bilateral massive PE and obstructive shock. The successful multidisciplinary management is described, highlighting the decisive role of percutaneous thrombectomy in achieving maternal-fetal survival by avoiding the hemorrhagic complications associated with traditional thrombolytic therapy.

## Case presentation

**Figure 1:**
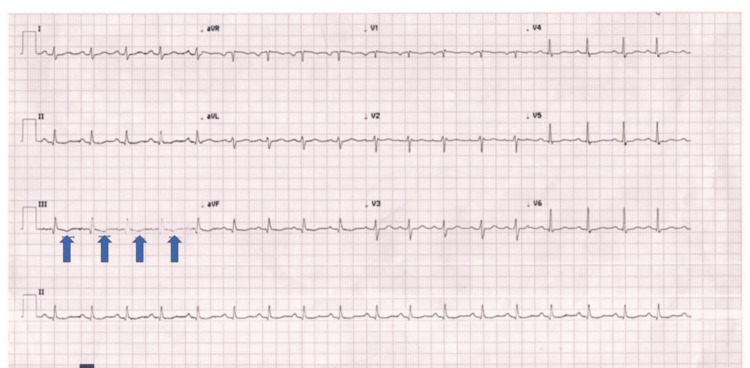
Electrocardiogram The results indicate sinus tachycardia, which is typically the most common electrocardiographic finding in this clinical setting. Inverted T waves are observed in lead III (blue arrows); however, the remaining criteria for the McGinn-White pattern (S1Q3T3 complex) are not fulfilled.

A 32-year-old multigravida woman (G5P4A1) at 29 weeks of gestation with a dichorionic diamniotic twin pregnancy and no significant past medical history presented to the emergency department with sudden-onset dyspnea and chest pain. On physical examination, she was alert and oriented, with sinus tachycardia at 125 beats per minute, blood pressure of 114/75 mmHg (mean arterial pressure of 88 mmHg), oxygen saturation of 77% on room air, and clinical signs consistent with obstructive shock. Given the abrupt presentation, clinical findings, and the prothrombotic state associated with twin pregnancy, the probability of PE was assessed using validated clinical prediction rules. The Wells score [5] indicated a moderate (intermediate) probability, consistent with the revised Geneva score [6]. Electrocardiography demonstrated sinus tachycardia (Figure 1).

**Figure 2:**
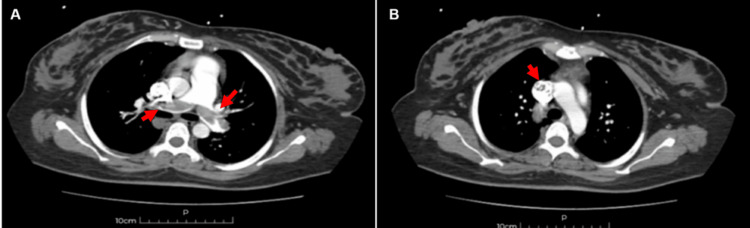
Computed tomography pulmonary angiography (CTPA) (A) CTPA image showing near-complete filling defects (red arrows) involving both main pulmonary artery branches. (B) CTPA image demonstrating a filling defect consistent with thrombus at the level of the superior vena cava (red arrow).

The gold standard in suspected PE is ventilation-perfusion (V/Q) scintigraphy or computed tomography pulmonary angiography (CTPA). In our case, chest CT angiography was performed (Figure 2), demonstrating bilateral PE involving both main pulmonary artery branches and inferior vena cava thrombosis extending into the right atrium.

Maternal severity and the risk of hemodynamic deterioration were further assessed using validated prognostic indices (BOVA model and simplified Pulmonary Embolism Severity Index, sPESI) to guide multidisciplinary decision-making. The BOVA prognostic score [7] was 5 points (Class III), corresponding to a high risk of mortality and complications of up to 42%. The sPESI was 2 points, also indicating a high risk of death and adverse outcomes [8,9].

Therapeutic anticoagulation with low-molecular-weight heparin was initiated, and the patient was admitted to the intensive care unit (ICU) under joint maternal-fetal and critical care management. Complementary laboratory studies were obtained and are presented in Table 1, including follow-up comparisons.

**Table 1:** Longitudinal summary of key laboratory parameters aPTT: activated partial thromboplastin time; HCO₃: bicarbonate; INR: international normalized ratio; PT: prothrombin time; NT-proBNP: N-terminal pro-B-type natriuretic peptide; PCO₂: partial pressure of carbon dioxide; SO₂: sulfur dioxide;

Parameter	Admission	24 Hours	48 Hours
pH	7.36	7.4	-
PCO₂ (mmHg)	34.3	37.1	-
HCO₃ (mmol/L)	19.1	22.9	-
Lactate (mg/dL)	24.4	11.2	-
SO₂ (%)	66.1	87.2	-
NT-proBNP (ng/mL)	125.7	906.7	1505
High-sensitivity troponin T (ng/mL)	42.9	67.9	71.2
aPTT (sec)	37.1	-	33.6
PT (sec)	11.3	-	13.1
INR	1.05	-	1.24
Hemoglobin (g/dL)	9.1	-	-
Leukocytes (10³/µL)	15.62	-	-
Platelets (10³/µL)	224	-	-

**Figure 3:**
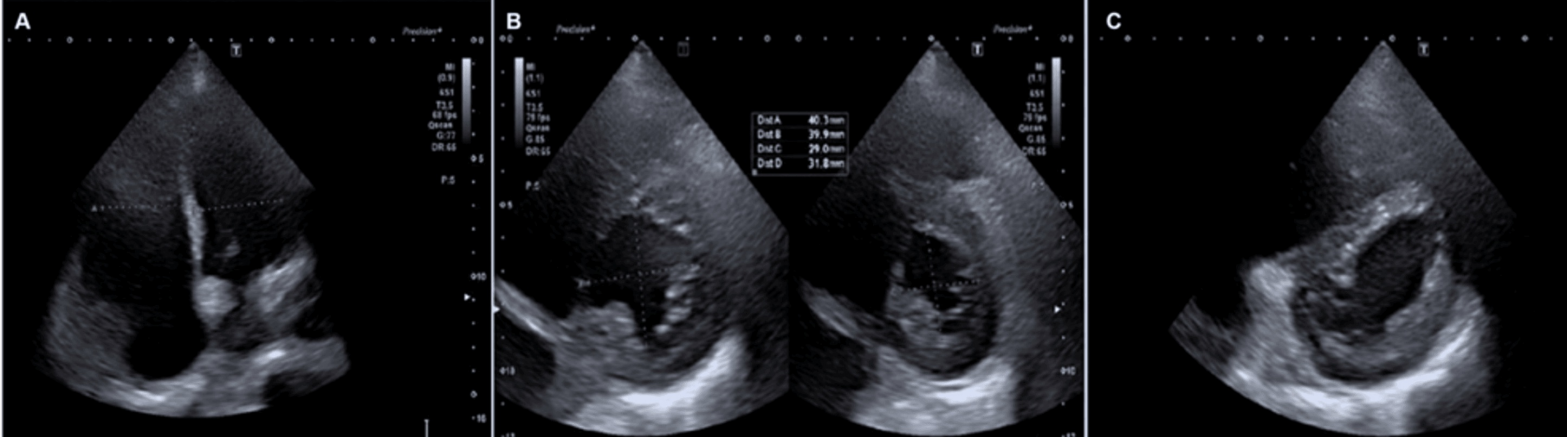
Transthoracic echocardiography (A) Apical four-chamber view demonstrating right ventricular dilation, with a right ventricle-to-left ventricle (RV/LV) ratio > 1. (B) Parasternal short-axis view showing interventricular septal displacement with reduced anteroposterior diameter of the left ventricle. (C) "D-sign" observed during systole, reflecting right ventricular pressure overload and septal flattening.

**Figure 4:**
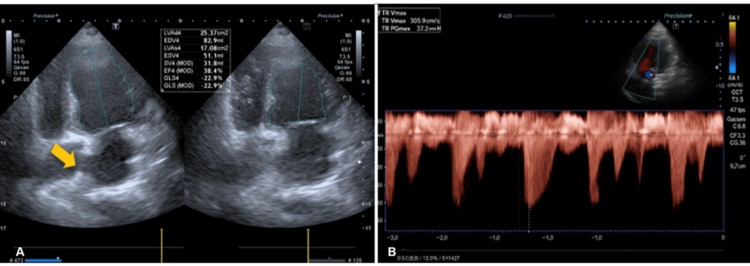
Follow-up transthoracic echocardiogram (A) Apical four-chamber view demonstrating reduced left ventricular systolic function with a left ventricular ejection fraction (LVEF) of 38.4% by Simpson's method. Global longitudinal strain (GLS) is reduced at -22.9%. An interatrial septal defect is visualized (yellow arrow). (B) Continuous-wave Doppler across the tricuspid valve demonstrating elevated tricuspid regurgitation maximum velocity (TR Vmax: 3.05 m/s), corresponding to a peak trans-tricuspid gradient of 37.2 mmHg, consistent with increased right ventricular systolic pressure.

Upon ICU admission, noninvasive respiratory support was initiated using a high-flow nasal cannula (HFNC). Bedside point-of-care ultrasound (POCUS) screening was performed (Figures 3-4), revealing early signs of right ventricular systolic dysfunction. Formal transthoracic echocardiography demonstrated right ventricular dilation (basal diameter: 46 mm, RV/LV: ratio >1), McConnell's sign, and systolic "D-sign," consistent with right-sided pressure overload. Left ventricular ejection fraction (LVEF) was 63% by the biplane Simpson method. Mean pulmonary artery pressure was 35 mmHg.

After 48 hours in the ICU, the patient developed progressive hemodynamic deterioration, including declining blood pressure, narrowed pulse pressure, prolonged capillary refill time (2.5 seconds), and worsening biomarkers (elevated troponin T and N-terminal pro-brain natriuretic peptide, NT-proBNP). Vasopressor support with norepinephrine was initiated, along with milrinone as an inodilator due to worsening right ventricular systolic dysfunction observed on repeat cardiac POCUS (Figure 4).

**Figure 5:**
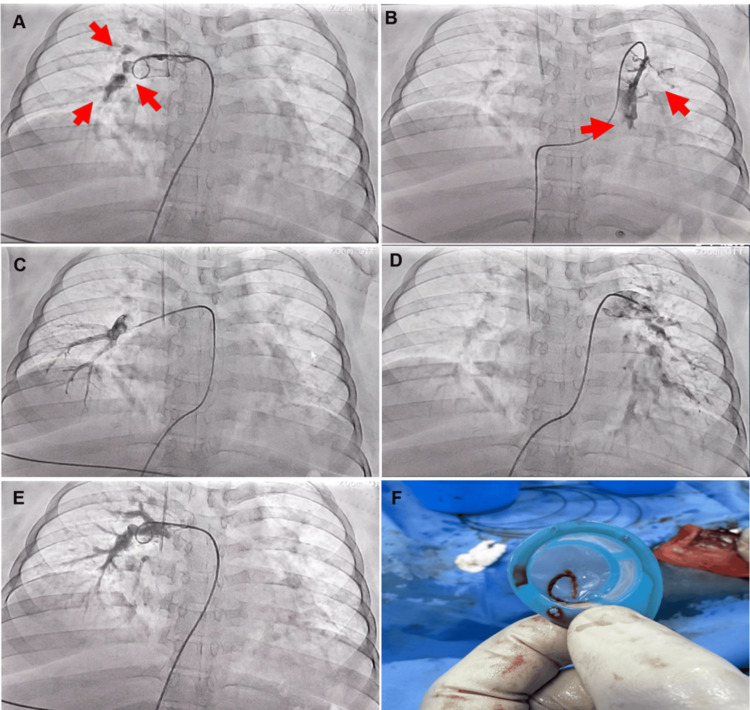
Computed tomography pulmonary angiography before and after mechanical thrombectomy Pre-thrombectomy: (A) Red arrow indicating extensive filling defects with >90% luminal narrowing at multiple sites involving the right main pulmonary artery and segmental branches, consistent with acute pulmonary embolism. (B) Red arrow demonstrating a filling defect at the level of the left main pulmonary artery, without contrast opacification, consistent with severe flow obstruction. Post-mechanical thrombectomy: (C) Restoration of contrast opacification in the right middle lobar artery, with filling of the medial, lateral, and apical segments. (D) Complete opacification of the left main pulmonary artery with adequate contrast passage into the left lower lobar artery, left upper lobar artery, and corresponding segmental branches. (E) Improved blood flow in the right main pulmonary artery, with complete filling of the middle lobar artery. (F) Extracted thrombus retrieved from the right main pulmonary artery.

In the setting of massive bilateral PE with obstructive shock and the absolute contraindication to systemic thrombolysis due to the high risk of obstetric hemorrhage and imminent preterm delivery, a multidisciplinary management strategy was established involving the ICU, interventional cardiology, and maternal-fetal medicine teams. A percutaneous mechanical thrombectomy via right femoral venous access was performed, achieving partial thrombus extraction and immediate restoration of pulmonary blood flow, with subsequent hemodynamic improvement and progressive increase in oxygen saturation (Figure 5).

During the first 24 hours post-procedure, the patient developed premature rupture of membranes and preterm labor, prompting emergency cesarean delivery. Two live neonates were delivered with Apgar scores of 8 and 9 and appropriate weight for gestational age. The postpartum course was uneventful, without hemorrhagic complications.

Therapeutic anticoagulation with low-molecular-weight heparin was continued and later transitioned to vitamin K antagonists following hemodynamic stabilization. Both the mother and the newborns had a favorable clinical evolution and were discharged in good condition.

## Discussion

Massive PE during pregnancy represents one of the most critical obstetric emergencies, with maternal mortality reaching alarming levels if not rapidly treated. This risk is further amplified in twin gestations, where the combination of an augmented hypercoagulable state and more pronounced venous stasis creates a particularly thrombogenic milieu compared with singleton pregnancies. Cohort data have shown that women with twin pregnancies exhibit higher fibrinogen levels and more marked activation of coagulation parameters throughout gestation than women with singleton pregnancies, confirming that coagulation is more enhanced in twin gestations, especially in the third trimester [10]. In parallel, echocardiographic and hemodynamic studies suggest that the greater uterine size in twin pregnancies results in higher central venous pressure and a smaller functional inferior vena cava diameter due to mechanical compression, thereby aggravating venous stasis relative to singleton pregnancies [11]. Taken together, these twin-specific physiological changes provide a plausible explanation for the rapid hemodynamic deterioration and impending obstructive shock observed in our patient once massive PE developed.

In the present case, the patient developed impending obstructive shock, posing an extreme therapeutic challenge due to the relative contraindication to systemic thrombolysis in the context of preterm pregnancy and high hemorrhagic risk. While anticoagulation remains the initial standard of care for PE during pregnancy, the patient's hemodynamic instability necessitated immediate reperfusion therapy.

The favorable maternal-fetal outcome in this case was largely attributable to rapid multidisciplinary decision-making involving specialists in intensive care, interventional cardiology, and maternal-fetal medicine. Percutaneous mechanical thrombectomy was selected as the most appropriate stabilization strategy compatible with ongoing pregnancy. Unlike systemic thrombolysis, which carries major bleeding rates of up to 6% and is associated with risks such as placental abruption and obstetric hemorrhage [12], percutaneous intervention allowed direct mechanical removal of the thrombotic burden, relieving right ventricular outflow obstruction and restoring pulmonary circulation without inducing systemic fibrinolysis or obstetric coagulopathy.

In our patient, this strategy resulted in rapid hemodynamic stabilization, reversal of acute right ventricular failure, and progressive improvement in oxygenation. Importantly, subsequent cesarean delivery performed 24 hours later due to premature rupture of membranes occurred in a controlled clinical context, free of pharmacologically induced coagulopathy, resulting in a hemorrhage-free postpartum course.

From a perinatal perspective, our findings reinforce the obstetric principle that effective maternal resuscitation constitutes the most reliable form of fetal resuscitation. While the patient was initially managed with an HFNC, the primary contribution of the mechanical thrombectomy was the rapid restoration of maternal hemodynamics and cardiac output by reducing right ventricular afterload. This hemodynamic stabilization was essential to restore critical uteroplacental perfusion, which was objectively reflected in the neonates' vigorous Apgar scores (8/9). Early intervention likely prevented irreversible fetal metabolic acidosis [13] and avoided the need for perimortem cesarean delivery, historically associated with extremely high neonatal morbidity and mortality due to intrapartum asphyxia [14].

Radiological safety remains a critical consideration in high-complexity obstetric interventions. While external fetal radiation shielding is a standard practice in CT to reduce scatter radiation, its specific benefit during fluoroscopy-guided procedures, such as mechanical thrombectomy, is less clearly established in the literature compared with technical dose-reduction strategies [15]. Contemporary radiology and obstetric guidance emphasize that the maternal benefit from early and accurate diagnosis and life-saving treatment generally outweighs the theoretical fetal risks associated with ionizing radiation, particularly when total fetal exposure remains well below the 50 mGy threshold under which no increase in congenital anomalies, growth restriction, or pregnancy loss has been demonstrated [16]. In this context, fetal protection in our case was prioritized through active technical modifications, strict beam collimation, and minimization of fluoroscopy time, while maternal survival was considered the primary determinant of fetal outcome.

Importantly, the current medical literature on endovascular reperfusion strategies in pregnancy remains limited, particularly in the context of multiple gestations. The most recent systematic review, which analyzed 76 reported cases of endovascular reperfusion in pregnancy, does not provide specific outcome data or management protocols for twin gestations [17]. Similarly, although successful use of mechanical thrombectomy in singleton pregnancies has been described, such as in the reports by Tébar-Márquez et al., evidence regarding its application in twin pregnancies remains absent [4].

To our knowledge, this report represents the first documented case of successful maternal survival with dual fetal survival in a third-trimester twin gestation treated with large-bore aspiration thrombectomy for massive PEl. Given the distinct hemodynamic and obstetric considerations associated with multiple gestations, extrapolation from singleton pregnancy data should be approached with caution. Consequently, this case provides clinically valuable evidence supporting the feasibility and potential safety of mechanical thrombectomy as a life-saving reperfusion strategy in complex twin pregnancies complicated by massive PE.

Finally, the successful outcome highlights the importance of structured multidisciplinary management in high-complexity centers such as Dr. Alejandro Dávila Bolaños Military School Hospital. Immediate activation of a PERT/Heart Team enabled rapid, coordinated decision-making guided by obstetric priorities, ultimately preserving both maternal and fetal survival. Our experience suggests that, in expert centers, mechanical thrombectomy should be considered early as a viable therapeutic option in life-threatening pregnancy-associated PE.

## Conclusions

Massive PE in twin pregnancy represents a highly lethal obstetric emergency requiring immediate therapeutic decisions aimed at preserving both maternal life and fetal viability. In this setting, percutaneous mechanical thrombectomy emerges as a safe and effective reperfusion alternative when systemic thrombolysis is contraindicated.

The favorable outcome observed highlights the value of a structured multidisciplinary approach in managing massive PE during pregnancy. Accordingly, the availability of PERT in high-complexity centers may optimize management and improve prognosis in these critical clinical scenarios.

## References

[1] Broder S, Paré PD (1996). Diagnosis and management of pulmonary embolism in pregnancy. Can Respir J.

[2] Madar H, Goffinet F, Seco A, Rozenberg P, Dupont C, Deneux-Tharaux C (2019). Severe acute maternal morbidity in twin compared with singleton pregnancies. Obstet Gynecol.

[3] Qadri S, Bilagi A, Sinha A, Connolly D, Murrin R, Bakour S (2024). Acute management of massive pulmonary embolism in pregnancy. Front Glob Womens Health.

[4] Tébar-Márquez D, Jurado-Román A, Jiménez-Valero S (2024). Percutaneous thrombectomy with FlowTriever system in pregnant women with high-risk pulmonary embolism and contraindications to systemic thrombolysis. J Endovasc Ther.

[5] Wells PS, Anderson DR, Rodger M (2001). Excluding pulmonary embolism at the bedside without diagnostic imaging: management of patients with suspected pulmonary embolism presenting to the emergency department by using a simple clinical model and D-dimer. Ann Intern Med.

[6] Le Gal G, Righini M, Roy PM, Sanchez O, Aujesky D, Bounameaux H, Perrier A (2006). Prediction of pulmonary embolism in the emergency department: the revised Geneva score. Ann Intern Med.

[7] Bova C, Sanchez O, Prandoni P, Lankeit M, Konstantinides S, Vanni S, Jiménez D (2014). Identification of intermediate-risk patients with acute symptomatic pulmonary embolism. Eur Respir J.

[8] Jiménez D, Aujesky D, Moores L (2010). Simplification of the pulmonary embolism severity index for prognostication in patients with acute symptomatic pulmonary embolism. Arch Intern Med.

[9] Righini M, Roy PM, Meyer G, Verschuren F, Aujesky D, Le Gal G (2011). The simplified pulmonary embolism severity index (PESI): validation of a clinical prognostic model for pulmonary embolism. J Thromb Haemost.

[10] Ren K, Wei Y, Qiao R, Shi H, Gong X, Zhao Y (2020). Changes in coagulation during twin pregnancies. Clin Appl Thromb Hemost.

[11] Nunez E, Huluta I, Gallardo Arozena M, Wright A, Nicolaides KH, Charakida M (2022). Maternal cardiac function in twin pregnancy at 19-23 weeks' gestation. Ultrasound Obstet Gynecol.

[12] Martillotti G, Boehlen F, Robert-Ebadi H, Jastrow N, Righini M, Blondon M (2017). Treatment options for severe pulmonary embolism during pregnancy and the postpartum period: a systematic review. J Thromb Haemost.

[13] Phelan JP, Pacheco LD, Foley MR, Saade GR, Dildy GA, Belfort MA (2018). Critical Care Obstetrics, 6th Edition. Critical Care Obstetrics. 6th ed. Wiley-Blackwell.

[14] American College of Obstetricians and Gynecologists (2017). Committee opinion no. 723: guidelines for diagnostic imaging during pregnancy and lactation. Obstet Gynecol.

[15] Marsh RM, Silosky M (2019). Patient shielding in diagnostic imaging: discontinuing a legacy practice. AJR Am J Roentgenol.

[16] Bhogal P, Aguilar M, AlMatter M, Karck U, Bäzner H, Henkes H (2017). Mechanical thrombectomy in pregnancy: report of 2 cases and review of the literature. Interv Neurol.

[17] Pititto GN, De Pascali F, Canale C, Squizzato A, Vedovati MC (2026). Endovascular reperfusion strategies for pregnancy-related pulmonary embolism: a systematic review. J Thromb Thrombolysis.

